# Fe_3_O_4_-Halloysite Nanotube Composites as Sustainable Adsorbents: Efficiency in Ofloxacin Removal from Polluted Waters and Ecotoxicity

**DOI:** 10.3390/nano12234330

**Published:** 2022-12-06

**Authors:** Doretta Capsoni, Paola Lucini, Debora Maria Conti, Michela Bianchi, Federica Maraschi, Beatrice De Felice, Giovanna Bruni, Maryam Abdolrahimi, Davide Peddis, Marco Parolini, Silvia Pisani, Michela Sturini

**Affiliations:** 1 Department of Chemistry, University of Pavia, 27100 Pavia, Italy; 2 C.S.G.I. (Consorzio Interuniversitario per lo Sviluppo dei Sistemi a Grande Interfase) & Department of Chemistry, Physical Chemistry Section, University of Pavia, 27100 Pavia, Italy; 3 Department of Environmental Science and Policy, University of Milan, 20133 Milan, Italy; 4 Institute of Structure of Matter, National Research Council (CNR), Monterotondo Scalo, 00015 Rome, Italy; 5 Dipartimento di Scienze, Università degli Studi Roma Tre, Via della Vasca Navale 84, 00146 Rome, Italy; 6 Department of Chemistry and Industrial Chemistry, University of Genova, 16146 Genova, Italy; 7 Department of Otorhinolaryngology, Fondazione IRCCS Policlinico San Matteo, 27100 Pavia, Italy

**Keywords:** magnetite-halloysite composites, magnetic sorbent materials, fluoroquinolone antibiotic, adsorption, wastewater treatment, magnetic remediation, emerging contaminants, ecotoxicity

## Abstract

The present work aimed at decorating halloysite nanotubes (HNT) with magnetic Fe_3_O_4_ nanoparticles through different synthetic routes (co-precipitation, hydrothermal, and sol-gel) to test the efficiency of three magnetic composites (HNT/Fe_3_O_4_) to remove the antibiotic ofloxacin (OFL) from waters. The chemical–physical features of the obtained materials were characterized through the application of diverse techniques (XRPD, FT-IR spectroscopy, SEM, EDS, and TEM microscopy, thermogravimetric analysis, and magnetization measurements), while ecotoxicity was assessed through a standard test on the freshwater organism *Daphnia magna*. Independently of the synthesis procedure, the magnetic composites were successfully obtained. The Fe_3_O_4_ is nanometric (about 10 nm) and the weight percentage is sample-dependent. It decorates the HNT’s surface and also forms aggregates linking the nanotubes in Fe_3_O_4_-rich samples. Thermodynamic and kinetic experiments showed different adsorption capacities of OFL, ranging from 23 to 45 mg g^−1^. The kinetic process occurred within a few minutes, independently of the composite. The capability of the three HNT/Fe_3_O_4_ in removing the OFL was confirmed under realistic conditions, when OFL was added to tap, river, and effluent waters at µg L^−1^ concentration. No acute toxicity of the composites was observed on freshwater organisms. Despite the good results obtained for all the composites, the sample by co-precipitation is the most performant as it: (i) is easily magnetically separated from the media after the use; (ii) does not undergo any degradation after three adsorption cycles; (iii) is synthetized through a low-cost procedure. These features make this material an excellent candidate for removal of OFL from water.

## 1. Introduction

In the current scenario of water shortage, there is an urgent need to favor water loops. For this purpose, preserving and guaranteeing water quality is mandatory, as reclaimed water can be directly reused and re-enter natural water bodies [1]. A critical aspect of water quality is represented by xenobiotics, such as heavy metals, dyes, pesticides, etc., detected in natural water bodies, also at trace levels, because of their recalcitrance in conventional wastewater treatment plants (WWTPs) [2]. In particular, pharmaceuticals and personal care products (PPCPs) have attracted the attention of the scientific community and civil society because of their widespread diffusion in the environment and their potential toxicity towards humans and ecosystems [3,4]. Although the current levels of PPCPs in aquatic ecosystems can be considered as low, they pose a severe threat for aquatic organisms because of their high biological activity and peculiar mechanism(s) of toxic action [4]. Among PPCPs, a remarkable concern is due to antibiotics whose presence in water ecosystems has been identified to affect natural microbial communities and to stimulate multi-resistant bacteria and antibiotic resistance genes, which pose serious risks to human and veterinary health [4,5]. To tackle the rising threats induced by the release of antibiotics, a recent action plan has proposed developing innovative strategies to reduce the diffusion of these emerging contaminants [6]. Over the last years, many research efforts have been made to develop sustainable and low-cost processes, easily implementable to conventional WWTPs and efficient in antibiotic removal from wastewater [7]. In this context, adsorption is a convenient method in terms of low energy consumption, reuse of the adsorbent material, no production of toxic by-products, and reduced waste production after treatment [8,9].

Many materials, both bare and functionalized, have been tested for water and wastewater decontamination, including activated carbon, nanomaterials, biopolymers, clays, agriculture and industrial wastes, and other natural sorbents [10,11,12,13].

The use of natural sorbents in the adsorption process [14,15,16] offers even more advantages, as they are abundant, low-cost, non-toxic, easy to modify, and competitive in water remediation compared to most conventional adsorbents [8,12,14].

Nanoclay materials surely fit the advantages mentioned above as sorbents to remove various pollutants, such as heavy metals, pesticides and antibiotics [17,18]. Among nanoclays, those displaying a tubular structure are even more intriguing, due to their additional properties related to the nanoscale dimension, cylindrical hollow form, and porosity. The halloysite nanotubes (HNTs) pertain to these nanoclays. Halloysites are aluminosilicates belonging to the kaolin group, with the chemical formula Al_2_Si_2_O_5_(OH)_4_ · *n* H_2_O. Two halloysite forms are reported in the literature, depending on the moles of hydrating molecules and the *d_001_* basal spacing: halloysite-(10 Å) is the di-hydrated form [19], and halloysite-(7 Å) is the anhydrous one. The latter form is the most common, due to the easy release of the halloysite water molecules at ambient conditions [20]. The halloysite structure is based on corner-sharing SiO_4_ tetrahedra sheets connected via oxygens to edge-sharing AlO_6_ octahedra ones [21,22]. The mismatch of the larger SiO_4_ tetrahedra and the smaller AlO_6_ octahedra accounts for the local stress on the atomic scale of the aluminosilicate layer, inducing its wrapping and the nanotubes’ morphology [22]. The nanotube typically displays lengths of 0.4–1 μm, an outer diameter of 20–200 nm, and an inner lumen diameter of 10–70 nm [23]. The siloxane (Si-O-Si) groups form the negatively charged outer surface, and the aluminol groups (-OH and Al-OH) form the positively charged inner one [24,25]. The peculiar physical and chemical features reported above make the HNTs suitable candidates for applications in various fields, including controlled drug release, nanotemplating, and adsorption. They are also employed as catalyst support and nanocomposites [26].

It is well known that the separation of the nanosorbent phase after pollutants removal is not a trivial challenge. A feasible and low-cost approach is to decorate the adsorbent material with magnetic nanoparticles to make it easily magnetically recovered. Some examples on the synthesis of halloysite–magnetite composites by co-precipitation, thermal decomposition, and solvothermal approaches are reported in the literature [27,28,29,30], and these materials are not yet investigated for water depollution.

Another key point to optimize before the application of nanomaterials in water remediation processes concerns the investigation of potential environmental and human risks associated with their use. The characterization of nanomaterials should have to include not only the assessment of any transformation occurring in environmental media, from its inclusion into a polluted site to the removal (or degradation) after the remediation of the target pollutant [31], but also the potential toxicity towards aquatic organisms. Ecotoxicology can provide useful tools to assess the risk related to nanomaterials and to select eco-friendly and sustainable ones for water remediation [32,33]. The application of standard and/or novel ecotoxicological tests completes the characterization of nanomaterials through the identification of possible toxicological targets and sheds light on the mechanism(s) of toxic action in aquatic species at different levels of the ecological hierarchy [34].

In the present study, we synthesized HNT/Fe_3_O_4_ nanocomposites by using three different approaches: co-precipitation, sol-gel, and hydrothermal. Each material was characterized by FT-IR spectroscopy, X-ray powder diffraction (XRPD), scanning electron and transmission electron microscopy (SEM and TEM), energy dispersive spectroscopy (EDS), thermogravimetric analysis (TGA), and magnetization measurements. Moreover, the magnetite and halloysite amount in each sample was evaluated by EDS, TGA, and magnetization data. Lastly, potential ecotoxicity of these materials towards aquatic organisms was tested on the freshwater Cladoceran *Daphnia magna* according to the *Daphnia* sp. Acute Immobilization Test, OECD 202 guideline (OECD, 2004). Adsorption properties and mechanism of each nanocomposite were investigated, and compared with the commercial halloysite. The antibiotic ofloxacin (OFL) was chosen as the target molecule to assess the adsorption efficiency of HNT/Fe_3_O_4_ nanocomposites for different reasons: (i) it is a very useful antibacterial agent belonging to the last class of antibiotics; (ii) it is largely detected in wastewaters and surface waters [3]; (iii) it is a recalcitrant to biological degradation [35]; (iv) it maintains a certain antibacterial activity after the first steps of its degradation [35]; (v) it has been used in our previous studies regarding both fluoroquinolones’ environmental fate and their removal by adsorption processes [36,37,38,39,40,41]. The suitability of three materials for OFL removal under environmental conditions, i.e., tap and river waters, and wastewater treatment plant (WWTP) effluent, was also verified.

## 2. Materials and Methods

### 2.1. Materials

All the chemicals employed were reagent grade or higher in quality. HNT, FeCl_3_·6H_2_O, FeSO_4_·7H_2_O, Fe(NO_3_)_3_·9H_2_O, ammonia solution (NH_3_ H_2_O), sodium acetate (CH_3_COONa), ethylene glycol (C_2_H_6_O_2_), ethanol (EtOH), glucose (C_6_H_12_O_6_), and OFL were purchased from Merck (Milano, Italy).

High-performance liquid chromatography (HPLC) gradient-grade acetonitrile (ACN) was purchased by VWR International (Milano, Italy), H_3_PO_4_ (85% *w*/*w*), and water for liquid chromatography/mass spectrometry (LC/MS) by Carlo Erba Reagents (Cornaredo, Milano, Italy).

### 2.2. Synthesis

Halloysite nanotubes–magnetite composites (HNT/Fe_3_O_4_) and magnetite alone (Fe_3_O_4_) were synthesized by co-precipitation, sol-gel, and hydrothermal routes, as follows.

Table 1 summarizes the synthesis approaches and the sample names.

#### 2.2.1. Co-Precipitation Procedure

The HNT/Fe_3_O_4_-C sample was synthesized following the procedure of Xie et al. [27]. An amount of 0.5 g of HNT was added to an aqueous solution of 4.32 mmol of FeCl_3_·6H_2_O and 2.16 mmol of FeSO_4_·7H_2_O. The suspension was heated at 60 °C under N_2_ flux, and an 8 M ammonia solution was added dropwise to reach pH 9–10. The suspension was further heated for 4 h at 70 °C, then the solid was magnetically recovered, washed three times, and dried for 3 h at 100 °C. The same procedure was applied to synthesize the Fe_3_O_4_ alone (sample Fe_3_O_4_-C), by omitting the addition of HNTs.

#### 2.2.2. Sol-Gel Procedure

The HNT/Fe_3_O_4_-SG sample was synthesized as reported by He et al. [29]. An amount of 1 g of HNT was dispersed in an ethanol solution containing 1.98 mmol of Fe(NO_3_)_3_·9H_2_O. The dispersion was sonicated, stirred 24 h at room temperature and dried for 24 h at 35 °C. An amount of 2 mL of ethylene glycol was added, and the sample was heated for 2 h at 400 °C under N_2_ flux (N_2_ 99.999%; flow rate: 3 L h^−1^; heating and cooling rate: 5 °C min^−1^). The same procedure was applied to synthesize the Fe_3_O_4_ alone (sample Fe_3_O_4_-SG), by omitting the addition of HNTs.

#### 2.2.3. Hydrothermal Procedure

HNT/Fe_3_O_4_-H sample was synthesized following the procedure of Tian et al. [30], with some modifications. The procedure consists of two hydrothermal steps: the former to prepare HNT enriched with a carbonaceous component, and the latter to decorate it with magnetite. An amount of 0.5 g of HNT was added to a glucose solution (10 g L^−1^) and magnetically stirred. The dispersion was poured into a Teflon-lined stainless-steel autoclave and heated for 48 h at 160 °C. The obtained product was washed 5 times in ethanol, centrifuged, and dried for 18 h at 60 °C under vacuum. An amount of 0.5 g of the final product was added to a solution containing 3 mmol of FeCl_3_·6H_2_O in ethylene glycol. After stirring for 24 h, 1.8 g of sodium acetate and 0.5 g of ethylene glycol were added, and the dispersion was poured into a Teflon-lined stainless-steel autoclave and heated for 8 h at 200 °C. The obtained magnetic composite was washed with distilled water and dried for 12 h at 80 °C. The procedure of the second step was also applied to synthesize the Fe_3_O_4_ alone (sample Fe_3_O_4_-H), by omitting the addition of HNTs.

#### 2.2.4. Characterization Techniques

X-ray powder diffraction measurements were performed using a Bruker D5005 diffractometer (Bruker, Karlsruhe, Germany) with the CuKα radiation, graphite monochromator, and scintillation detector. The patterns were collected in the 7–80° two-theta angular range, step size of 0.03°, and a counting time of 20 s/step. A silicon low-background sample holder was used.

FT-IR spectra were obtained with a Nicolet FT-IR iS10 Spectrometer (Nicolet, Madison, WI, USA) equipped with ATR (attenuated total reflectance) sampling accessory (Smart iTR with ZnSe plate) by co-adding 32 scans in the 4000–650 cm^−1^ range at 4 cm^−1^ resolution.

Thermogravimetric measurements were performed by a TGA Q5000 IR apparatus interfaced with a TA 5000 data station (TA Instruments, Newcastle, DE, USA). The samples were scanned at 10 °C min^−1^ under nitrogen flow (45 mL min^−1^) in the 20–850 °C temperature range. Each measurement was repeated at least three times.

The specific surface area and porosity were investigated by N_2_ adsorption using the BET method in a Sorptomatic 1990 Porosimeter (Thermo Electron, Waltham, MA, USA).

SEM measurements were performed using a Zeiss EVO MA10 (Carl Zeiss, Oberkochen, Germany) Microscope, equipped with an Energy Dispersive Detector for the EDS analysis. The SEM images were collected on gold-sputtered samples. HR-SEM images were taken from an FEG-SEM Tescan Mira3 XMU. Samples were mounted onto aluminum stubs using double sided carbon adhesive tape and were then made electrically conductive by coating in vacuum with a thin layer of Pt. Observations were made at 25 kV with an In-Beam SE detector at a working distance of 3 mm.

TEM micrographs were carried out on a JEOL JEM-1200 EX II (JEOL Ltd., Tokio, Japan) microscope operating at 100 kV high voltage (tungsten filament gun) and equipped with a TEM CCD camera Olympus Mega View III (Olympus soft imaging solutions (OSIS) GmbH, from 2015 EMSIS GmbH, Munster, Germany) with 1376 × 1032 pixel format. The samples were prepared by drop-casting the solution on nickel grids formvar/carbon coated.

Dynamic light scattering (DLS)—Nicomp 380 ZLS (Particle Sizing Systems, Lakeview Blvd. Fremont, CA, USA) was used. For analyses, samples were diluted 1:10 in MilliQ water. The main parameters set up were: channel 10, intensity 100 kHz, temperature 23 °C, viscosity 0.933 cPoise, and a liquid index of refraction 1.333. The values considered at the end of the analyses were: mean diameter (nm), standard deviation, and Zeta potential (mV).

To investigate the magnetic behavior of the materials, field dependence of magnetization was investigated using a vibrating sample magnetometer (VSM Model 10–Microsense) equipped with an electromagnetic producing magnetic field in the range ±2 T.

### 2.3. Adsorption Experiments and Analytical Measurements

#### 2.3.1. Adsorption and Kinetic Experiments

OFL adsorption on HNT/Fe_3_O_4_-C, HNT/Fe_3_O_4_-SG, HNT/Fe_3_O_4_-H, and commercial HNT was studied by a batch method. For adsorption equilibrium experiments, 20 mg of each material was suspended in 10 mL of tap water spiked with OFL in the range of 25–200 mg L^−1^. Flasks were wrapped with aluminum foil to prevent light-induced drug decomposition and shaken for 24 h at room temperature with an orbital shaker. Subsequently, the suspensions were magnetically separated, and the supernatants were filtered (0.22 μm) and analyzed by UV-vis spectrophotometer at 287 nm to determine the antibiotic concentration in solution at equilibrium (*C_e_*). The adsorbed OFL amount at equilibrium (*q_e_*, mg g^−1^) was calculated by Equation (1):

(1) $$q_{e}=\frac{(C_{0}-C_{e})\cdot V}{m}$$

where *C*_0_ is the initial OFL concentration (mg L^−1^), *C_e_* is the drug concentration in solution at equilibrium (mg L^−1^), *V* is the volume of the solution (L), and *m* is the amount of the sorbent material (*g*).

For the kinetic experiments, 20 mg of each material were suspended in 10 mL of 20 mg L^−1^ OFL tap water solution. Falcon tubes, wrapped with aluminum foil, were shaken by a roller shaker and, at selected times, the adsorbent was magnetically treated. Then, a few mL of the supernatant were collected, filtered (0.22 μm) in a quartz cuvette, and analyzed by a UV spectrophotometer at 287 nm. The analyzed solution was recovered to keep the suspension volume constant for all experiments. The adsorbed OFL amount at time *t* (*q_t_*, mg g^−1^) was calculated as (Equation (2)):

(2) $$q_{t}=\frac{(C_{0}-C_{t})\cdot V}{m}$$

where *C*_0_ is the initial OFL concentration (mg L^−1^), *C_t_* is the drug concentration in solution at time *t* (mg L^−1^), *V* is the volume of the solution (L), and *m* is the amount of the sorbent material (*g*).

All experiments were performed in duplicate. The thermodynamic and kinetic parameters were estimated by dedicated software (OriginPro, Version 2019b. OriginLab Corporation, Northampton, MA, USA).

The well-known Langmuir’s and Freundlich’s isotherm models were applied to fit the experimental data. The Langmuir model (Equation (3)) describes the adsorption process that takes place on specific homogeneous sites and in a monolayer on the material surface:

(3) $$q_{e}=\frac{q_{m}K_{L}C_{e}}{1+K_{L}C_{e}}$$

where *K_L_* is the Langmuir constant and *q_m_* is the monolayer saturation capacity.

The Freundlich model defines non-ideal adsorption on the heterogeneous surface, and Equation (4) expresses it:

(4) $$q_{e}=K_{F}C_{e}^{1/n}$$

where *K_F_* is the empirical constant indicative of adsorption capacity, and *n* is the empirical parameter representing the adsorption intensity.

The time-dependent data were fitted by pseudo-first-order (Equation (5)) and pseudo-second-order kinetic (Equation (6)) models:

(5) $$q_{t}=q_{e}\left(1-e^{k_{1}t}\right)$$

(6) $$q_{t}=\frac{q_{e}^{2}k_{2}t}{1+q_{e}k_{2}t}$$

where *q_t_* and *q_e_* are the drug adsorbed amount at time *t* and equilibrium, respectively, and *k*_1_ and *k*_2_ are the pseudo-first-order and the pseudo-second-order rate constants.

#### 2.3.2. Analytical Measurements

For OFL analysis at mg L^−1^, a UV-vis UVmini-1240 spectrophotometer (Shimadzu Corporation) was used. The instrument was set at 287 nm, corresponding to the maximum OFL absorption. Calibration in the range of 1–10 mg L^−1^ yielded optimal linearity (R^2^ > 0.9988). The quantification limit was 0.8 mg L^−1^.

HPLC system consisting of a pump Series 200 (Perkin Elmer, Milano, Italy) equipped with a vacuum degasser and a programmable fluorescence detector (FD) was used for OFL analysis at µg L^−1^. The fluorescence excitation/emission wavelengths selected were 280/450 nm. Fifty µL of each sample were filtered (0.22 µm nylon syringe filter) and injected into a 250 × 4.6 mm, 5 µm Ascentis RPAmide (Supelco-Merck Life Science, Milano, Italy) coupled with a similar guard-column. The mobile phase was 25 mM H_3_PO_4_—ACN (85:15), and the flow rate 1 mL min^−1^. Calibration in the range 1–20 µg L^−1^ yielded optimal linearity (R^2^ > 0.9988). The quantification limit was 0.9 µg L^−1^.

### 2.4. Acute Toxicity Tests with Daphnia magna

The potential acute toxicity of the different materials, i.e., HNT, Fe_3_O_4_-C, and HNT/Fe_3_O_4_-C, was tested on the freshwater Cladoceran *Daphnia magna* according to the *Daphnia* sp. Acute Immobilization Test, OECD 202 guideline (OECD, 2004). Adult *Daphnia magna* individuals were cultured (30 individuals/L) in a commercial mineral water (San Benedetto^®^) under controlled laboratory conditions reported elsewhere [42]. Five replicates containing ten daphnids (i.e., <24 h old individuals) each were performed per each experimental condition, including control. In detail, daphnids were exposed for 48 h at 20 ± 0.5 °C and 16 h light: 8 h dark photoperiod under static, non-renewal conditions to 0.2 g L^−1^ of the materials. A single concentration mimicking the amount of residues in waters after depollution treatment was tested. This concentration reflected the amount of each material used in the experiments aimed at investigating their capability in the removal of OFL. The viability of individuals was tested after 24 and 48 h of exposure. Individuals were considered dead when they did not swim for over 15 s after a slight stirring of the solutions. After checking for viability, all the individuals were observed under a Leica Microsystem EZ4 Stereoscopic microscope to check for the ingestion of materials by daphnids.

## 3. Results and Discussion

First, structure, morphology, composition, magnetic behavior, adsorption capacity, and adsorption kinetics of the magnetic HNT composites and the commercial HNT were investigated. Then the materials were tested under environmental conditions to remove the antibiotic OFL chosen as being representative of emerging contaminants. In addition, their potential ecotoxic effects, along with reusability, were evaluated.

### 3.1. Morphological, Structural, and Magnetic Characterization

**Figure 1:**
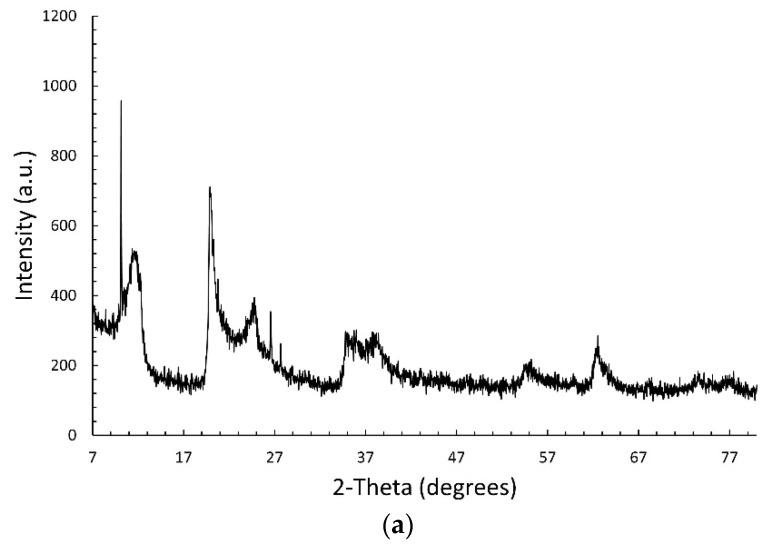

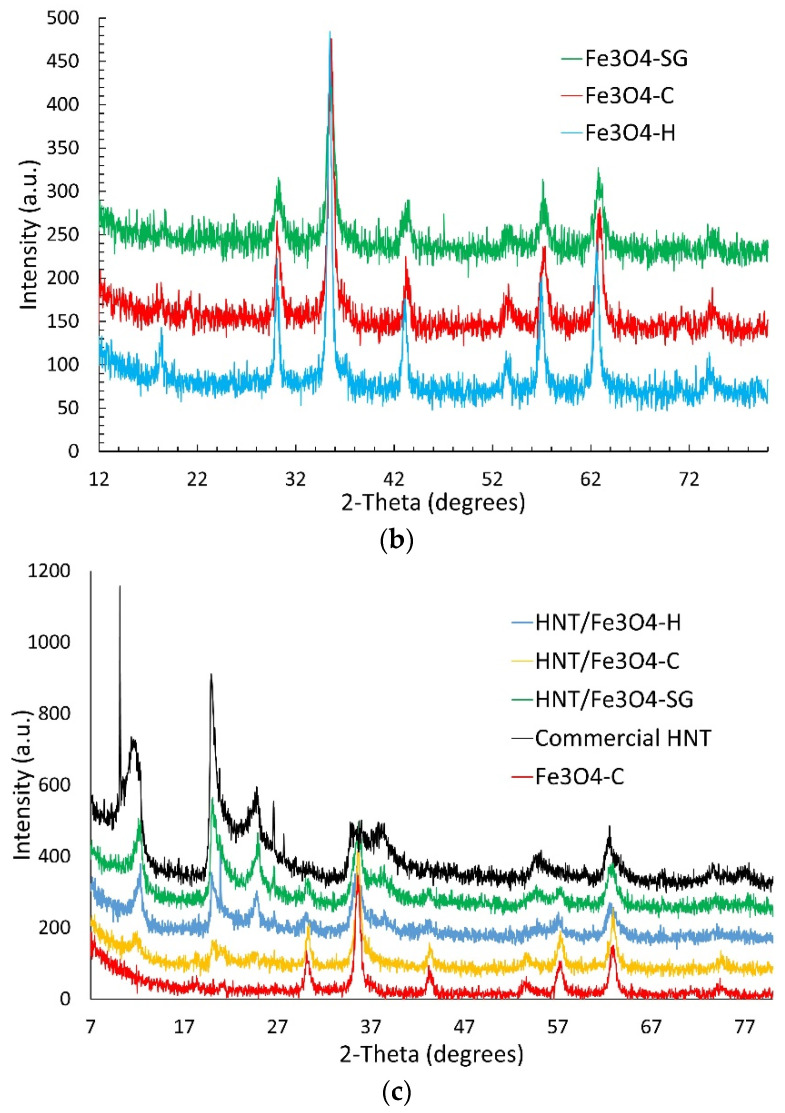
XRPD patterns of (**a**) commercial HNT, (**b**) Fe_3_O_4_, and (**c**) HNT/Fe_3_O_4_ composites, Fe_3_O_4_ and HNT.

Figure 1a shows the XRPD pattern of the commercial halloysite. It compares to those reported in the literature [27,30,43,44] and deposited in JCPDS database (PDF# 028-1487). The peak detected at about 12° corresponds to the *d_001_* basal spacing of 7.35 Å, peculiar of the anhydrous form (halloysite-(7 Å)). The (002) reflection is observed at about 24°. The peaks at 20° and 62.8° are typical of halloysites with nanotubular morphology [44,45]. No peaks are detected at about 8.8°, assigned to the *d_001_* basal spacing of the di-hydrated halloysite (halloysite-(10 Å)). This is consistent with the easy loss of the interlayer water molecules near room temperature [46]. The very sharp reflections observed at 10.1, 26.6, and 27.3° are attributed respectively to the small amount of kaolinite 1A (PDF# 074-1786), quartz (PDF# 046-1045), and rutile (PDF# 021-1276); these impurity phases are often detected in halloysite clay minerals.

Figure 1b displays the XRPD pattern of the Fe_3_O_4_ samples obtained by the three synthetic routes. The 2-theta reflection positions fairly agree with those expected for the magnetite structure (PDF# 088-0315). The iron oxide phase has been successfully synthesized, and no impurity phases are detected within the detection limit of the technique. The iron oxide samples are nanocrystalline: a crystallite size of 10, 13, and 8 nm was calculated for Fe_3_O_4_-C, Fe_3_O_4_-SG, and Fe_3_O_4_-H samples by applying the Scherrer equation to the 311 reflection.

Figure 1c displays the diffraction pattern of the magnetite–halloysite composites. The diffraction patterns of the commercial halloysite and the Fe_3_O_4_-C sample (chosen as reference for the magnetic phase), are also shown for comparison. The three composite samples display the peaks of both the magnetite and the halloysite phases, thus confirming the successful formation of the magnetite–halloysite adduct. An investigation of the magnetite crystallite size in the composites by applying the Scherrer equation could not be carried out, due to the strong overlap of the 311 reflection of the magnetite phase to the broad peaks of halloysite in the 33–40° 2 theta range. Nonetheless, the comparable peaks broadening of the magnetite phase in the composites and in the Fe_3_O_4_ samples suggests nanocrystalline magnetite is obtained also in the HNT/Fe_3_O_4_ samples. The peaks’ intensity of halloysite and magnetite in the composite samples returns an idea on the phases amount in each sample. The peaks’ intensity of halloysite decreases and Fe_3_O_4_ increases progressively from HNT/Fe_3_O_4_-SG to HNT/Fe_3_O_4_-H and HNT/Fe_3_O_4_-C, suggesting that the magnetite and halloysite amounts in the composite samples depend on the synthesis route.

**Figure 2:**
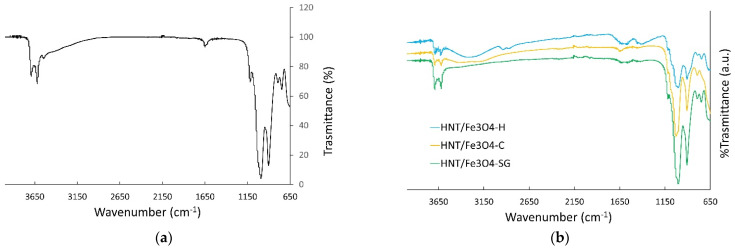
FT-IR spectra of (**a**) the commercial halloysite, and (**b**) the HNT/Fe_3_O_4_ composites.

The FT-IR spectra of the commercial halloysite and the HNT/Fe_3_O_4_ composites are shown in Figure 2. The spectrum of the commercial HNT well compares to the literature ones [27,29,30,43]. The bands centered at about 3622 and 3707 cm^−1^ are attributed to the stretching vibrations of the Al-OH of the HNT inner surface, while the small peaks at about 3545 and 1641 cm^−1^ to the stretching and banding of the H_2_O molecules in the interlayer. This result puts into evidence the possible presence of small amount of the hydrated form (halloysite-(10 Å)) in the commercial halloysite, below the detection limit of XRPD. The bands at about 1031, 794, and 689 cm^−1^ are attributed to the Si-O stretching modes, the one at about 918 cm^−1^ to the Al-OH ones. In the FT-IR spectra of the HNT/Fe_3_O_4_ composites (Figure 2b), all the halloysite bands are detected. As for the Fe_3_O_4_ phase, only one broad band centered at about 3435 cm^−1^ attributed to OH-bending of hydroxyl groups was observed [43]. This broad band was not detected in the HNT/Fe_3_O_4_-SG sample, displaying a high amount of halloysite and a few magnetites (see XRPD results).

**Figure 3:**
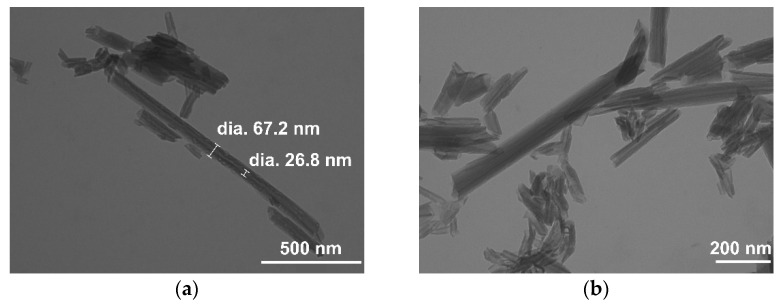
TEM images of the commercial halloysite sample at magnifications of (**a**) 75 kX and (**b**) 100 kX.

The SEM images of the commercial HNT are shown in Figure S1a,b. The sample displayed 2–10 μm agglomerates of nanotubular particles, better highlighted in TEM micrographs (Figure 3a,b). The nanotubes exhibited an external diameter of 60–70 nm, a lumen of 20–30 nm, and variable length, from a few hundred nanometers to 1–2 μm. The DLS results showed a bimodal particle size distribution. The mean particle size is reported in Table S1.

Figure S2 shows the SEM micrographs of the HNT/Fe_3_O_4_ composites synthesized by co-precipitation (Figure S2a,b), hydrothermal (Figure S2c,d), and sol-gel (Figure S2e,f) routes. All the composites displayed micrometric nanotubular particles, whose morphology well compares to the HNT sample one (Figure S1a,b). In addition, nanometric rounded aggregates, possibly due to the magnetite phase, were observed on the nanotubes surface and between the nanotubes, interconnecting them; they were mainly detected in the HNT/Fe_3_O_4_-C sample (Figure S2a,b) which was richer in magnetite, as suggested by XRPD and FT-IR results.

**Figure 4:**
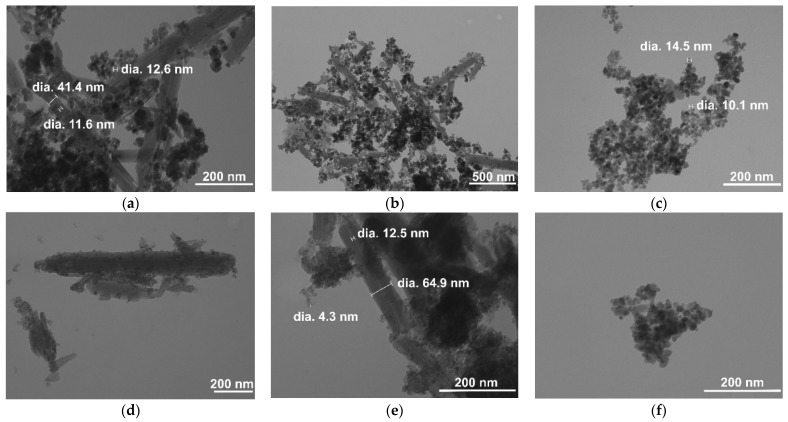

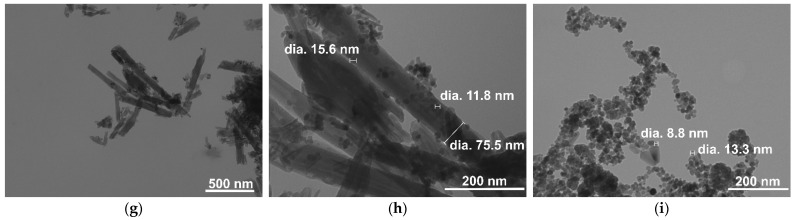
TEM images of the HNT/Fe_3_O_4_ and magnetite samples. HNT/Fe_3_O_4_-C at magnifications of (**a**) 150 kX and (**b**) 50 kX: Fe_3_O_4_-C (**c**) at 150 kX; HNT/Fe_3_O_4_-H at (**d**) 100 kX and (**e**) 200 kX; Fe_3_O_4_-H (**f**) at 200 kX; HNT/Fe_3_O_4_-SG at (**g**) 50 kX and (**h**) 200 kX; Fe_3_O_4_-SG (**i**) at 150 kX.

Figure 4 displays the TEM images of the HNT/Fe_3_O_4_ composites and Fe_3_O_4_ samples synthesized by co-precipitation (Figure 4a–c), hydrothermal (Figure 4d–f), and sol-gel (Figure 4g–i) routes. Independent of the applied synthesis, both rounded Fe_3_O_4_ nanometric particles and halloysite nanotubes were observed in the HNT/Fe_3_O_4_ composites. Noteworthy, the Fe_3_O_4_ amount was high in the HNT/Fe_3_O_4_-C sample (Figure 4a,b); it covered the nanotubes’ surface, but also formed aggregates linking the nanotubes. This was also slightly observed in the HNT/Fe_3_O_4_-H sample. The Fe_3_O_4_ agglomerates were mainly observed on the tips of nanotubes. As reported by Tian et al. [30], the synthetic strategy based on the use of glucose in the first step favored the formation of carbon/organic groups on the HNT surface and on the tip of nanotubes, acting as nucleation centers for the Fe_3_O_4_ nanoparticles. As for the HNT/Fe_3_O_4_-SG sample, it displayed a lower amount of magnetite (see XRPD and FT-IR results), and the Fe_3_O_4_ nanoparticles only decorated the nanotubes’ surface. The size and shape of the magnetite nanoparticles in the composites (about 10 nm) well compared to the Fe_3_O_4_ samples (Figure 4c,f,i) for the Fe_3_O_4_-C, Fe_3_O_4_-H, and Fe_3_O_4_-SG respectively), and fairly agreed with the crystallite size evaluated by XRPD data. In both the magnetite and composite samples, the Fe_3_O_4_ nanoparticles aggregate; particle size distribution was evaluated by DLS analysis and reported in Table S1. The Fe_3_O_4_-C sample displayed wide particle size distribution. The HNT/Fe_3_O_4_-C and NHT/Fe_3_O_4_-SG samples displayed particle size >900 nm, slightly similar to the larger ones of the commercial halloysite. Instead, the HNT/Fe_3_O_4_-H composite displayed lower particle size. To better characterize the tendency of particles to aggregate and to investigate particles’ surface charge changes, zeta-potential was evaluated. Commercial HNT exhibits a negative zeta-potential of −31.77 mV; this value confirms that the outer nanotube surface is negatively charged and is in good agreement with the literature data [47].

The Fe_3_O_4_-C sample (chosen as reference of the magnetite samples) exhibits a zeta-potential of −7.16 mV, comparable to the literature values [48]; this value is not sufficient to achieve a stable suspension, and justifies particle aggregation (see TEM and DLS results).

Zeta-potential values of −36.36, −12.89 and −112.02 mV are obtained for HNT/Fe_3_O_4_-C, HNT/Fe_3_O_4_-SG and HNT/Fe_3_O_4_-H composites. The sample prepared by the hydrothermal process displays the most negative zeta-potential value; this may be due to the carbonaceous component (see TEM results and Section 3.2.) and explains the improved stability of the suspension and the lower mean particle size, as shown by DLS results.

The EDS analysis was applied to display the distribution map of halloysite and magnetite in each composite sample and to evaluate the weight percentage. Figures S3–S5 show the distribution maps of Al, Fe, and Si for the HNT/Fe_3_O_4_-C, HNT/Fe_3_O_4_-H, and HNT/Fe_3_O_4_-SG samples. Independently of the synthetic route, Al and Si were detected in the same areas. The Fe distribution was rather homogeneous in the sol-gel and hydrothermal samples (Figures S4 and S5, respectively), but also in some regions in which Fe prevails were detected. In the co-precipitation composite, Fe prevailed in areas poor in Al and Si, thus confirming the presence of magnetite aggregates connecting the halloysite particles.

From the EDS analysis, the Al, Si, and Fe atomic percentages were evaluated. Al:Si:Fe molar ratios of 5.25:5.15:20.33, 5.11:5.03:4.62, and 12.36:13.35:3.46 were obtained for the HNT/Fe_3_O4-C, HNT/Fe_3_O_4_-H, and HNT/Fe_3_O_4_-SG samples, respectively. According to the halloysite chemical formula, equimolar values of Al and Si were detected in each sample. The molar ratios obtained by EDS were used to calculate halloysite and magnetite weight percentage in each composite: the results are shown in Table 2.

**Figure 5:**
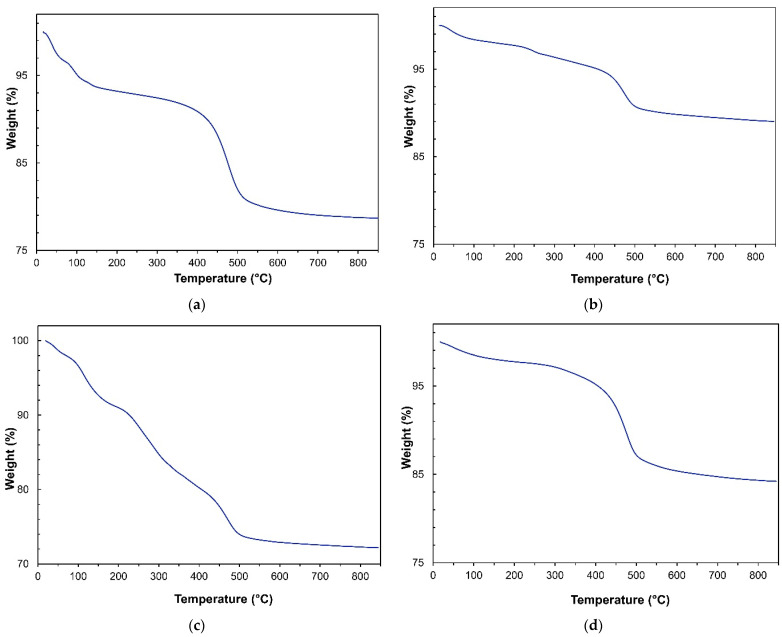
TGA curves of (**a**) commercial HNT (**b**) HNT/Fe_3_O_4_-C, (**c**) HNT/Fe_3_O_4_-H, and (**d**) HNT/Fe_3_O_4_-SG samples.

The halloysite amount in the HNT/Fe_3_O_4_ composites was also calculated by thermogravimetric analyses. The thermograms of commercial HNT and composites are shown in Figure 5. The halloysite TG curve (Figure 5a) well compared to the literature data [27]. The mass loss detected at low temperature (below 250 °C) was ascribed to the release of physisorbed water molecules. The steep mass loss observed at about 450 °C gave more insight, as it is due to the dehydroxylation process of the structural Al-OH groups of the aluminosilicate layers. A weight loss of 13.95% was calculated from halloysite stoichiometry. The mass loss detected in the commercial HNT was about 14.60%, in fair agreement with the calculated value. Figure 5b–d show the thermograms of the HNT/Fe_3_O_4_-C, HNT/Fe_3_O_4_-H, and HNT/Fe_3_O_4_-SG samples, respectively. Different mass losses were detected at low temperature (below 250 °C), depending on the amount of the physisorbed water, then a sample-dependent steep mass loss occurs at about 450 °C. As reported by Xie et al. [27], this mass loss can be compared to the HNT sample one (14.60%) to evaluate the halloysite weight percentage in each composite. The results are reported in Table 2; the halloysite weight percentages well compared to the values obtained by EDS analysis.

**Figure 6:**
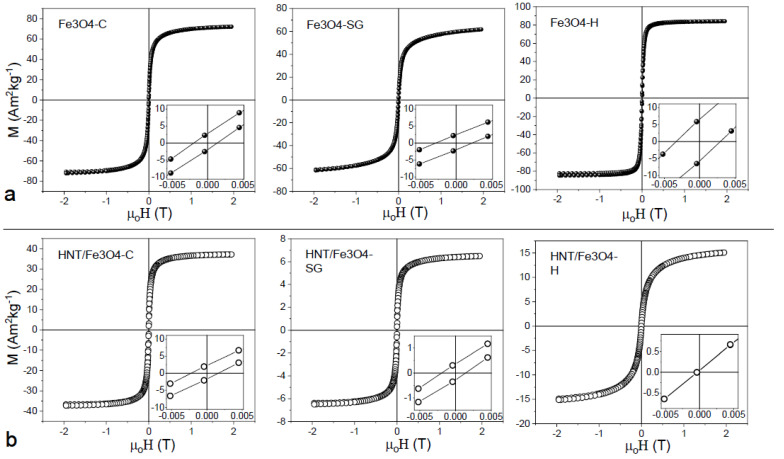
Field dependence of magnetization at 300 K for the (**a**) bare Fe_3_O_4_ nanoparticles synthetized by coprecipitation (Fe_3_O_4_-C) sol-gel (Fe_3_O_4_-SG) and hydrothermal methods (Fe_3_O_4_-H) and (**b**) HNTs/Fe_3_O_4_ nanocomposites. The insets are zoom of the coercive field region.

Field dependence of magnetization was investigated for all the samples at 300 K (Figure 6a,b).

For bare nanoparticles prepared with co-precipitation and sol-gel synthesis methods (Figure 6a), negligible value of reduced remanence magnetization (Mr/Ms) and small value of coercivity were obtained (Table 3), suggesting that at 300 K most of the nanoparticles were in a superparamagnetic state and just a small fraction of nanoparticles showed a quasi-static behavior. While the zero coercivity in the nanoparticles synthesized with the hydrothermal procedure indicated that all nanoparticles were in a supermagnetic state. Fe_3_O_4_-C and Fe_3_O_4_-SG samples showed a weak non-saturating character at high field, with respect to the Fe_3_O_4_-H sample. Due to the small difference in size between the samples, a non-saturating character showed by samples prepared by sol-gel and co-precipitation techniques can be ascribed to an increase in surface anisotropy, probably due to the presence of magnetic disorder (i.e., canted spin) [49,50] at the particles’ surface. This hypothesis was also confirmed by the decrease in M_S_ in SG and C samples. All the HNT nanocomposites showed a decrease in M_S_ with respect to bare nanoparticles in qualitative agreement with TGA and EDS measurements. This behavior confirmed that the amount of magnetic phase decreases along the order Fe_3_O_4_-C, Fe_3_O_4_-SG, and Fe_3_O_4_-H. From a quantitative point of view, if the agreement among magnetization measurements, TGA and EDS, was pretty good for Fe_3_O_4_-SG and Fe_3_O_4_-H, a difference was observed for Fe_3_O_4_-C nanocomposite. In particular, the particles prepared by co-precipitation looked to decrease their M_S_ when prepared as nanocomposites. This can be ascribed to a decrease in nanoparticles’ crystallinity that can be observed in the co-precipitation synthesis with respect to hydrothermal and sol-gel syntheses [51,52].

It is well known that the adsorption capacity of the materials is strictly related to their specific surface area [53]. The BET method was applied to investigate the specific surface area of the commercial halloysite and the three HNT/Fe_3_O_4_ composites. The values of 58.20, 57.66, 52.15, and 54.56 m^2^ g^−1^ were obtained for the commercial HNT, HNT/Fe_3_O_4_-C, HNT/Fe_3_O_4_-H, and HNT/Fe_3_O_4_-SG samples, respectively. The pore specific volume was also evaluated, and values of 0.19, 0.26, 0.16, and 0.27 cm^3^ g^−1^ were obtained. These results suggest that the deposition of the magnetite nanoparticles on the nanotubular halloysite surface did not affect the halloysite surface area and pore volumes. The obtained values fairly agreed with the literature data for halloysite nanotubes (surface areas: 22.1–81.6 m^2^ g^−1^; pore volumes: 0.09–0.25 cm^3^ g^−1^) [22].

### 3.2. Preliminary Adsorption Experiments

Before starting the adsorption experiments, control samples (20 mg HNT/Fe_3_O_4_ or HNT, 10 mL tap water), not containing OFL, were shaken for 24 h at room temperature. Then, the supernatants were magnetically separated for the pH measurement and analyzed by UV-vis spectrophotometer and HPLC-FD to check the instrumental baseline.

A pH value of 7.7–7.8, similar to that of natural waters, was measured in all samples, thus no additional pH adjustment was performed.

The background noise level was satisfactory for the commercial HNT, HNT/Fe_3_O_4_-C, and HNT/Fe_3_O_4_-H. On the contrary, HNT/Fe_3_O_4_-SG was rinsed with EtOH in an ultrasonic bath for 10 min, centrifuged for 5 min at 4000 rpm, separated, and dried at 50 °C for 1.5 h. The washing step was repeated twice to obtain a good signal-to-noise ratio.

### 3.3. Isotherm and Kinetic Studies

The behavior of the three magnetic HNT composites was evaluated through thermodynamic and kinetic experiments carried out under controlled conditions (see Section 2.3.1) and compared with the commercial HNT.

Adsorption isotherms are commonly used to describe the adsorption process in terms of maximum uptake and the relationship between the amount of adsorbed analyte (*q_e_*) and its concentration in solution at equilibrium (*C_e_*).

To fit the experimental data, the Langmuir and Freundlich models were considered.

**Figure 7:**
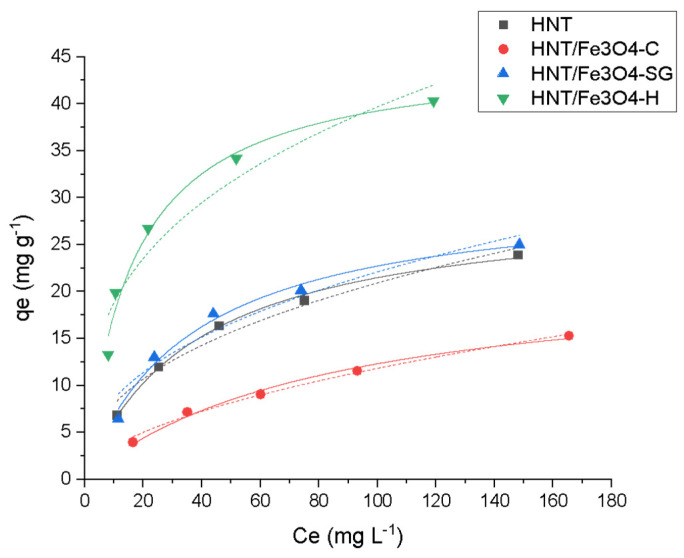
Adsorption profiles Langmuir (—) and Freundlich (…) for Ofloxacin (OFL) on HNT (■ black), HNT/Fe_3_O_4_-C (● red), HNT/Fe_3_O_4_-H (▲ blue) and HNT/Fe_3_O_4_-SG (▼ green) (Experimental conditions: Sorbent 20 mg, 10 mL OFL tap water solution from 25 to 200 mg L^−1^, RSD < 10%).

As shown in Figure 7, the Langmuir model gave the best fitting of the experimental data.

Figure 7 shows that all materials were able to adsorb the antibiotic, although the maximum adsorption capacities were quite different. In detail, the highest value, 45 mg g^−1^, was obtained for HNT/Fe_3_O_4_-H, while the lowest value was obtained for HNT/Fe_3_O_4_-C, which was equal to 23 mg g^−1^. The HNT/Fe_3_O_4_-SG sample had an intermediate value of 31 mg g^−1^, close to the commercial HNT (30 mg g^−1^). This trend can be due to both the different amount of HNT present in the samples, ranging from about 30% in HNT/Fe_3_O_4_-C to more than 80% in HNT/Fe_3_O_4_-SG (see Table 2), and to the possible presence of some carbonaceous component related to the glucose added during HNT/Fe_3_O_4_-H synthesis. In fact, as reported by Tian et al. [30], the carbon/organic groups formed on the HNTs not only favor the Fe_3_O_4_ nanoparticle nucleation, but also may improve the analyte adsorption. On the contrary, no difference in the adsorption mechanism was observed among all materials. The Langmuir model, which describes a monolayer coverage, gives the best fitting of the experimental data, as confirmed by the good correlation coefficient R^2^ and χ^2^ values.

The experimental *q_max_* values of HNT/Fe_3_O_4_-C, HNT/Fe_3_O_4_-H, and HNT/Fe_3_O_4_-SG were in agreement with the calculated ones, and fell within the OFL adsorption range reported in the literature for other clays, i.e., 3.2 mg g^−1^ on kaolinite [54], 160.8 mg g^−1^ on calcined Verde-lodo bentonite clay [55]).

The isothermal parameters calculated by dedicated software are listed in Table 4.

**Figure 8:**
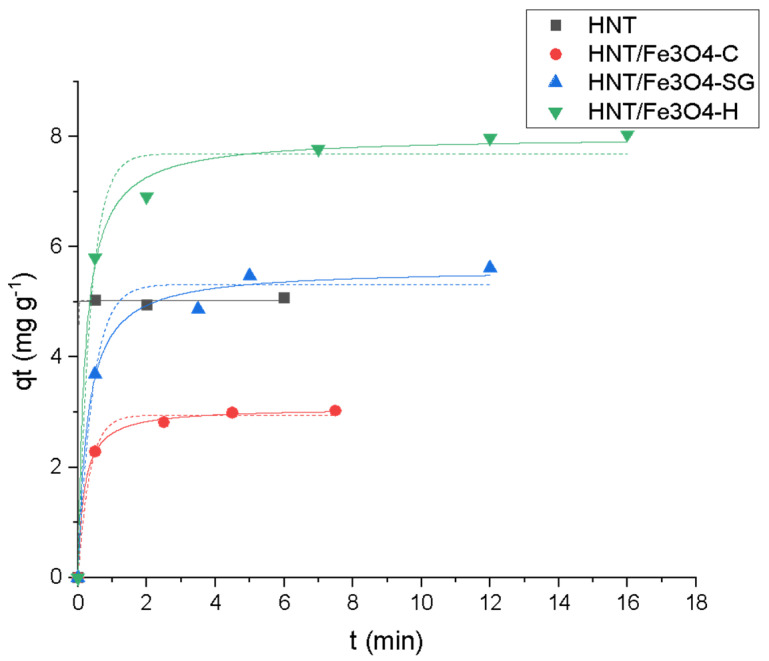
Kinetic profiles (pseudo-first-order (—), pseudo-second-order (…)) for OFL onto HNT (■ black), HNT/Fe_3_O_4_-C (● red), HNT/Fe_3_O_4_-H (▲ blue) and HNT/Fe_3_O_4_-SG (▼ green) (Experimental conditions: sorbent 20 mg, 10 mL tap water, OFL initial concentration 20 mg L^−1^, RSD < 10%).

Concerning the kinetic aspect, quantitative adsorption occurred in less than five minutes in the presence of all the magnetic composites. As shown in Figure 8, a satisfactory fitting is obtained by applying the pseudo-second-order model, thus, considering a chemisorption process. For commercial HNT, the adsorption was instantaneous, thus, it was not possible to discriminate between the two models.

The calculated kinetic parameters are shown in Table 5.

#### 3.3.1. Ofloxacin Removal from Real Waters Samples

Magnetic HNTs were also tested under environmental conditions, i.e., µg L^−1^ OFL concentration, tap and river waters, WWTP effluent (see Table S2 for the physicochemical parameters).

An amount of 20 mg of each material was suspended in 10 mL of each water sample, river water and WWTP effluent samples spiked with 10 μg L^−1^ OFL (*C*_0_) and shaken for 24 h. Then, the suspensions were magnetically separated and the supernatants were filtered on a 0.22 μm nylon syringe filter before HPLC-FD analysis to quantify the drug content (*C_e_*).

The removal efficiency (*R*%) was calculated according to Equation (3):

(7) $$R\%=\frac{C_{0}-C_{e}}{C_{0}}\times 100$$

where *C*_0_ is the initial OFL concentration and *C_e_* is the OFL concentration in solution at the equilibrium.

**Figure 9:**
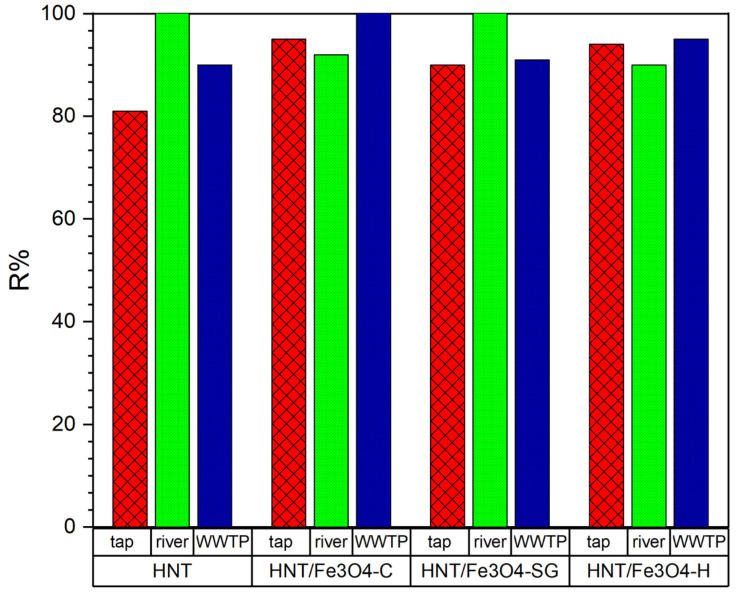
OFL removal (%) from tap and river water samples and effluent from WWTPS with HNT, HNT/Fe_3_O_4_-C, HNT/Fe_3_O_4_-H, and HNT/Fe_3_O_4_-SG (Experimental conditions: sorbent 20 mg, 10 mL tap water, OFL initial concentration 10 µg L^−1^, *n* = 3, RSD < 10%).

The obtained results were reported in Figure 9.

The investigated HNT/Fe_3_O_4_ composites gained an antibiotic removal ≥90% despite different aqueous matrix constituents and other potential contaminants. The different amount of Fe_3_O_4_ in each composite did not affect the adsorption process; on the contrary, the Fe_3_O_4_ percent in HNT/Fe_3_O_4_-C, higher than in HNT/Fe_3_O_4_-H and HNT/Fe_3_O_4_-SG, favored its complete magnetic recovery from the media after the use with no additional centrifugation step.

#### 3.3.2. Reusability and Post-Use Characterization of HNT/Fe_3_O_4_-C

Among the investigated magnetic HNTs, the HNT/Fe_3_O_4_-C sample ensured a quantitative OFL removal in different real water samples and excelled for its magnetic properties. For these reasons, its reusability was explored.

The HNT/Fe_3_O_4_-C sample was suspended in 10 mL tap water containing OFL 10 µg L^−1^. After 1 h, HNT/Fe_3_O_4_-C was magnetically separated, and the supernatant was analyzed by HPLC-FD. Then the recovered sorbent material was suspended for a second time in 10 mL tap water samples containing OFL 10 µg L^−1^. After 1 h contact, the suspended material was magnetically separated, and the OFL concentration in the solution was measured. A third cycle was carried out following the same procedure.

**Figure 10:**
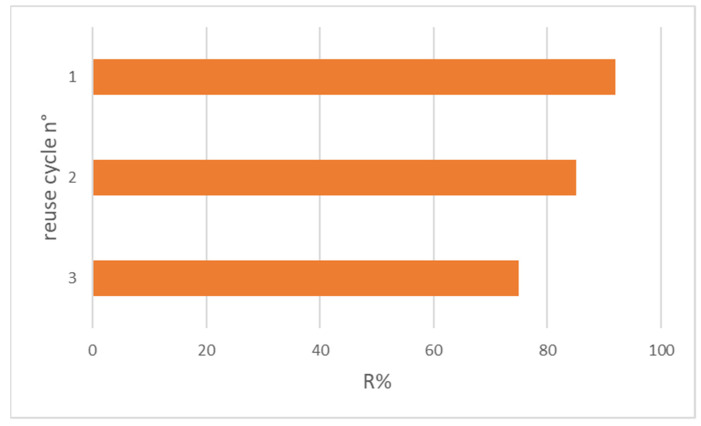
OFL removal after three reuse cycles with HNT/Fe_3_O_4_-C (Experimental conditions: sorbent 20 mg, 10 mL tap water, OFL initial concentration 10 µg L^−1^).

Figure 10 shows the adsorbed OFL percentage after each adsorption cycle. The adsorbed antibiotic amount slightly decreased from 95% after the first use to 75% after the third one.

This trend may be ascribed to a small loss of material during its magnetic separation from the sample solution and not to matrix interference, as XRPD analysis demonstrates.

The recovered sorbent material after three adsorption cycles was analyzed by XRPD and compared to the synthesized HNT/Fe_3_O_4_-C sample. The two diffraction patterns (Figure S6) are really comparable, confirming the sorbent material does not undergo degradation processes with use.

#### 3.3.3. Acute Toxicity Test with *Daphnia magna*

For the toxicity test, a single concentration, equal to 0.2 g L^−1^ of HNT, Fe_3_O_4_, and HNT/Fe_3_O_4_-C was tested. This concentration reflected a potential residual amount of each material in waters after depollution treatment.

**Figure 11:**
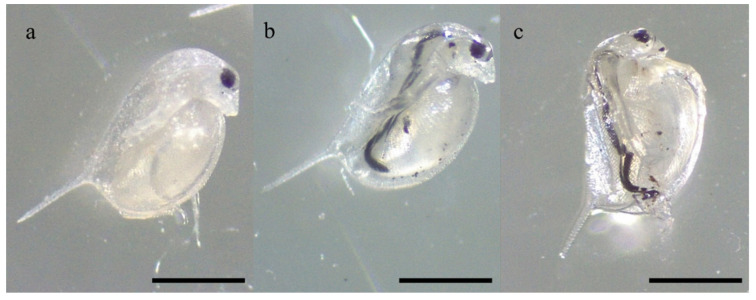
Individuals of *D. magna* showing their digestive tract full of HNT (**a**), Fe_3_O_4_ (**b**), and HNT/Fe_3_O_4_-C (**c**) after 48 h of exposure to 0.2 g L^−1^ (10 mg/50 mL) for each material. Scale bar = 500 µm.

All the individuals efficiently ingested the administered materials over 48 h of exposure (Figure 11), as shown by their presence in the digestive tract of exposed individuals.

No mortality occurred in the control group. Despite the ingestion of all the materials, the 48 h exposure to 0.2 g L^−1^ of HNT and HNT/Fe_3_O_4_-C did not induce the mortality of any daphnid, while the viability of the individuals included in the Fe_3_O_4_ experimental group was slightly decreased compared to the corresponding control, accounting for the 96 ± 9%.

## 4. Conclusions

In the present work, magnetic halloysite nanotubes were successfully synthesized by three different approaches: co-precipitation, hydrothermal, and sol-gel method. The applied characterization techniques demonstrate that the nanometric-sized Fe_3_O_4_ (diameter of about 10 nm) were formed and connected to the HNT particles. Magnetic phase abundance depended on the synthetic route and was evaluated by EDS and TGA analyses, as well as by magnetization data. Thermodynamic and kinetic experiments suggested that HNT/Fe_3_O_4_ composites can be considered as performing materials for ofloxacin adsorption. All the investigated samples were able to quantitatively reduce the antibiotic concentration under realistic conditions and, more interestingly, the sample obtained by the co-precipitation synthetic approach—the most cost-effective—was also easily magnetically removed from the media after treatment and reused for three cycles with no degradation. The ecotoxicity test performed on the freshwater organism *D. magna* completed the characterization of this adsorbent material and confirmed that it might be safely applied in water depuration processes.

## Figures and Tables

**Table 1:** Scheme of the synthesis procedures and samples names.

Synthesis Procedure	Sample	Sample Name
Coprecipitation	magnetite halloysite g–magnetite	Fe_3_O_4_-C HNT/Fe_3_O_4_-C
Sol-gel	magnetite halloysite–magnetite	Fe_3_O_4_-SG HNT/Fe_3_O_4_-SG
Hydrothermal	magnetite halloysite–magnetite	Fe_3_O_4_-H HNT/Fe_3_O_4_-H

**Table 2:** Halloysite and magnetite weight percentages evaluated by EDS, TGA, and magnetization data.

Sample	Halloysite (wt%)			Magnetite (wt%)		
	EDS	TGA	Magnetization	EDS	TGA	Magnetization
HNT/Fe_3_O_4_-C	30	29	12	70	71	88
HNT/Fe_3_O_4_-H	65	65	68	35	35	32
HNT/Fe_3_O_4_-SG	85.5	83	93	14.5	17	7

**Table 3:** Saturation magnetization M_S_, reduce remanence magnetization (Mr/M_S_) and coercive field (µ_0_H_C_) of Fe_3_O_4_-C, Fe_3_O_4_-SG, Fe_3_O_4_-H, HNT/Fe_3_O_4_-C, HNT/Fe_3_O_4_-SG, and HNT/Fe_3_O_4_-H samples.

Samples	Ms (Am^2^ kg^−1^)	Mr/Ms	µ_0_H_C_ (Oe)
Fe_3_O_4_-C	70 (5)	0.03 (2)	16 (2)
HNT/Fe_3_O_4_-C	37 (4)	0.06 (2)	19 (4)
Fe_3_O_4_-SG	56 (3)	0.04 (2)	25 (4)
HNT/Fe_3_O_4_-SG	6 (2)	0.05 (2)	16 (3)
Fe_3_O_4_-H	83 (3)	0.06 (3)	32 (5)
HNT/Fe_3_O_4_-H	13 (5)	0	0

**Table 4:** Isotherm parameters for OFL adsorption onto HNT, HNT/Fe_3_O_4_-C, HNT/Fe_3_O_4_-H, and HNT/Fe_3_O_4_-SG.

Adsorption Model	Isotherm Parameters	HNT	HNT/Fe_3_O_4_-C	HNT/Fe_3_O_4_-SG	HNT/Fe_3_O_4_-H
Langmuir	*q_m_* (mg g^−1^)	29.6 (8)	23 (2)	31 (2)	45 (2)
	*K_L_* (L mg^−1^)	0.026 (2)	0.012 (2)	0.028 (4)	0.063 (9)
	R^2^	0.9970	0.9910	0.9881	0.9840
	χ^2^	0.1739	0.2218	0.7893	2.5004
Freundlich	*K_F_* (mg g^−1^) (L mg^−1^)^1/*n*^	3.1 (6)	1.1 (1)	3 (1)	9 (2)
	1/*n*	0.42 (4)	0.53 (3)	0.41 (7)	0.33 (5)
	R^2^	0.9734	0.9931	0.9381	0.9304
	χ^2^	1.5239	0.1712	4.1164	10.844

**Table 5:** Kinetic parameters for OFL adsorption onto HNT, HNT/Fe_3_O_4_-C, HNT/Fe_3_O_4_-H, and HNT/Fe_3_O_4_-SG.

Kinetic Model	Kinetic Parameter	HNT	HNT/Fe_3_O_4_-C	HNT/Fe_3_O_4_-SG	HNT/Fe_3_O_4_-H
Pseudo-first order	*q_e_* (mg g^−1^)	5.02 (4)	2.95 (5)	5.3 (2)	7.7 (2)
	*k*_1_ (min^−1^)	124	3.0 (3)	2.4 (4)	2.8 (5)
	R^2^	0.9996	0.9961	0.9854	0.9839
	χ^2^	0.0041	0.0086	0.1052	0.1931
Pseudo-second order	*q_e_* (mg g^−1^)	5.02 (6)	3.08 (3)	5.6 (2)	8.0 (1)
	*k*_2_ (g mg^−1^ min^−1^)	3888	1.8 (2)	0.7 (2)	0.61 (9)
	R^2^	0.9996	0.9992	0.9926	0.9965
	χ^2^	0.0041	0.0017	0.0531	0.0418

## Data Availability

The data presented in this study are available on request from the corresponding author.

## Supplementary Materials

The following supporting information can be downloaded at: https://www.mdpi.com/article/10.3390/nano12234330/s1, Figure S1: SEM images of the commercial halloysite at (a) 9 kX and (b) 200 kX.; Figure S2: SEM images of the HNT/Fe_3_O_4_ composites. (a,b): HNT/Fe_3_O_4_-C sample; (c) and (d): HNT/Fe_3_O_4_-H sample; (e,f): HNT/Fe_3_O_4_-SG sample. Magnification: 9 kX (left) and 200 kX (right); Figure S3: (a) investigated area and distribution maps of (b) Al, (c) Fe and (d) Si elements of the HNT/Fe_3_O_4_-C sample; Figure S4: (a) investigated area and distribution maps of (b) Al, (c) Fe and (d) Si elements of the HNT/Fe_3_O_4_-H sample; Figure S5: (a) investigated area and distribution maps of (b) Al, (c) Fe and (d) Si elements of the HNT/Fe_3_O_4_-SG sample; Figure S6: X-ray diffraction pattern of the HNT/Fe_3_O_4_-C sample as-prepared (black line) and after three cycles of OFL recover (red line); Table S1: Mean particle size and intensity determined by DLS analysis; Table S2: Physico-chemical characterization of tap and river water samples, and WWTP effluent.
