# Supplementary material for: Fe3O4-Halloysite Nanotube Composites as Sustainable Adsorbents: Efficiency in Ofloxacin Removal from Polluted Waters and Ecotoxicity
Source: Nanomaterials (Basel). 2022 Dec 6;12(23):4330. doi: 10.3390/nano12234330 (PMC9739226; doi:10.3390/nano12234330)
Supplement: Supplementary file 1 [file nanomaterials-12-04330-s001.zip › nanomaterials-2045163-supplementary.pdf]

Fe_3O_4-Halloysite Nanotube Composites as Sustainable Adsorbents: Efficiency in Ofloxacin Removal from Polluted Waters and Ecotoxicity

Doretta Capsoni [1,2], Paola Lucini [1,2], Debora Maria Conti [1,2], Michela Bianchi [1], Federica Maraschi [1], Beatrice De Felice [3], Giovanna Bruni [1,2], Maryam Abdolrahimi [4,5], Davide Peddis [4,6], Marco Parolini [3], Silvia Pisani [7] and Michela Sturini [1,2,*]

1 Department of Chemistry, University of Pavia, 27100 Pavia, Italy; doretta.capsoni@unipv.it (D.C.); paola.lucini01@universitadipavia.it (P.L.); deboramaria.conti01@universitadipavia.it (D.M.C.); michela.bianchi02@universitadipavia.it (M.B.); federica.maraschi@unipv.it (F.M.); giovanna.bruni@unipv.it (G.B.)
2 C.S.G.I. (Consorzio Interuniversitario per lo Sviluppo dei Sistemi a Grande Interfase) & Department of Chemistry, Physical Chemistry Section, University of Pavia, 27100 Pavia, Italy
3 Department of Environmental Science and Policy, University of Milan, 20133 Milan, Italy; beatrice.defelice@unimi.it (B.D.F.); marco.parolini@unimi.it (M.P.)
4 Institute of Structure of Matter, National Research Council (CNR), Monterotondo Scalo, 00015 Rome, Italy; maryam.abdolrahimi@uniroma3.it (M.A.); davide.peddis@unige.it (D.P.)
5 Dipartimento di Scienze, Università degli Studi Roma Tre, Via della Vasca Navale 84, 00146 Roma, Italy
6 Department of Chemistry and Industrial Chemistry, University of Genova, 16146 Genova, Italy
7 Department of Otorhinolaryngology, Fondazione IRCCS Policlinico San Matteo, 27100 Pavia, Italy; s.pisani@smatteo.pv.it
* Correspondence: michela.sturini@unipv.it; Tel.: +39-0382-987347

SEM IMAGES

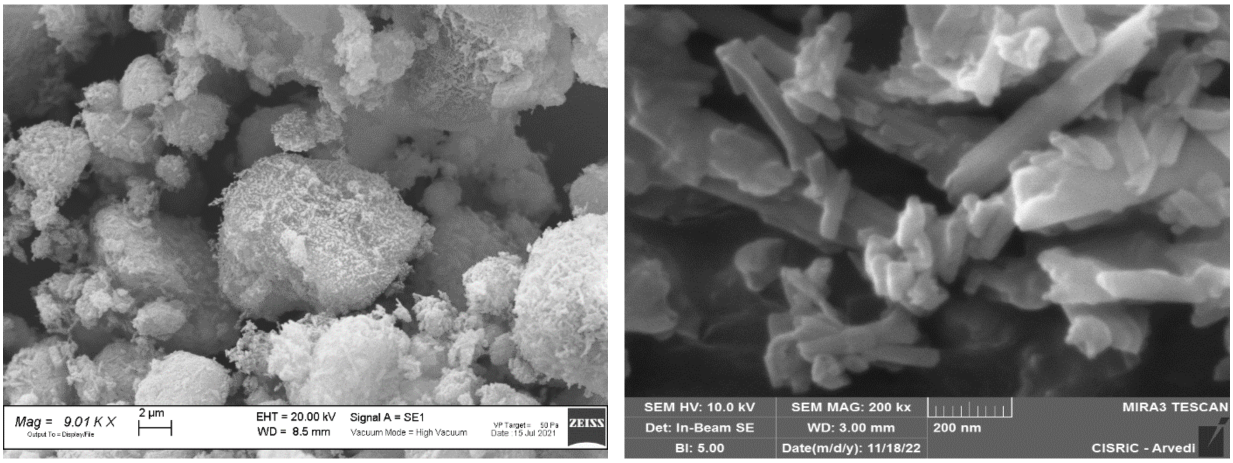

(a) (b)

Figure S1 – SEM images of the commercial halloysite at (a) 9 kX and (b) 200 kX.

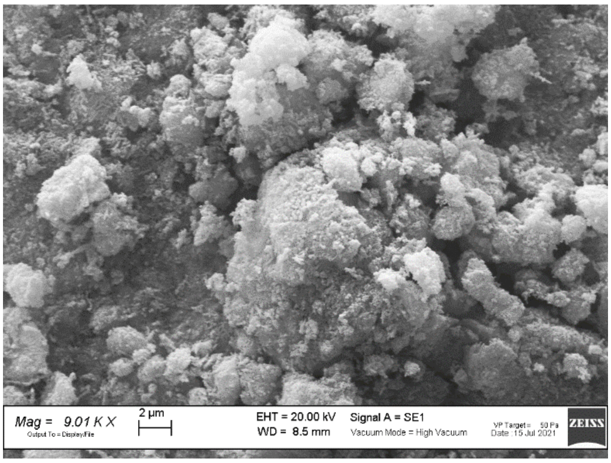

(a)

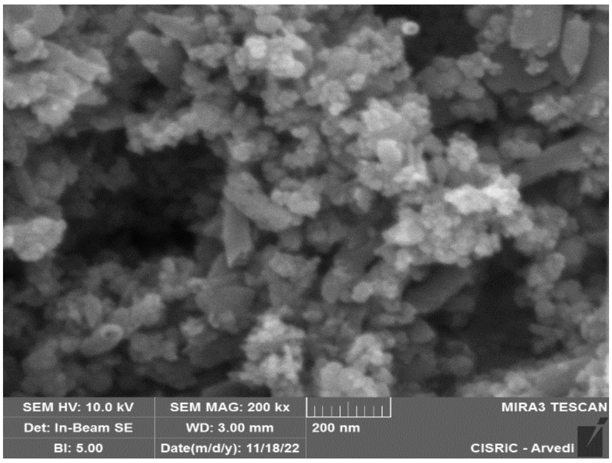

(b)

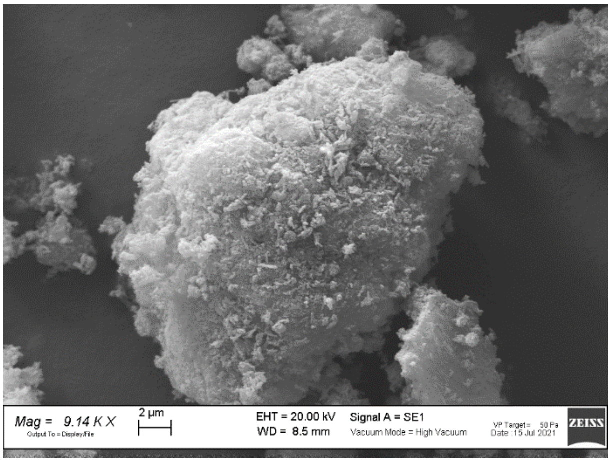

(c)

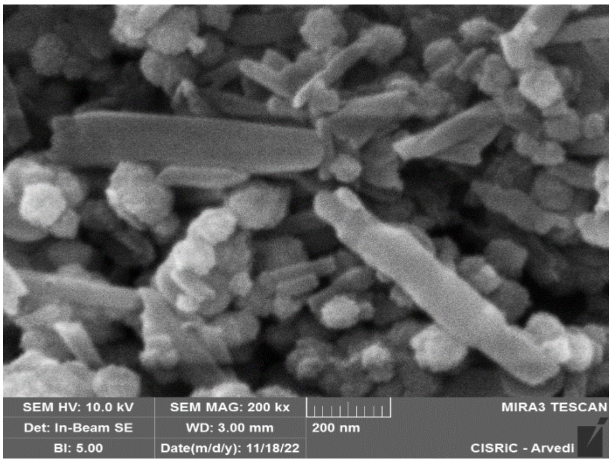

(d)

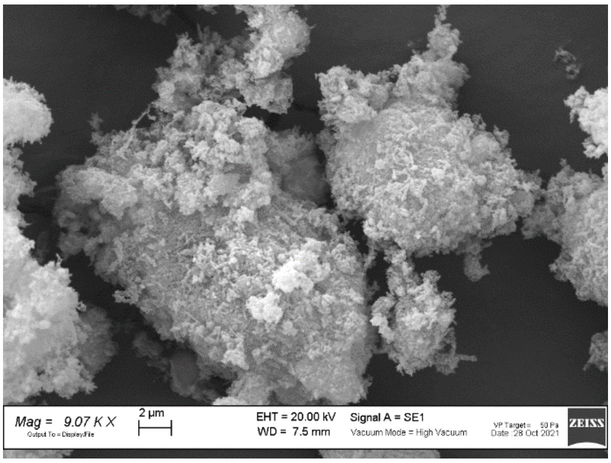

(e)

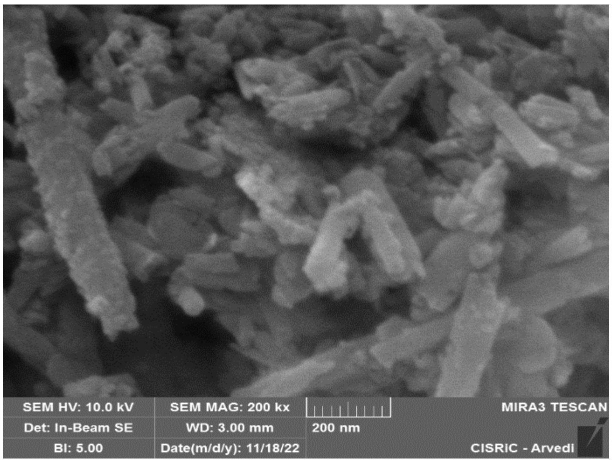

(f)

Figure S2 – SEM images of the HNT/Fe_3O_4 composites. (a) and (b): HNT/Fe3O4-C sample; (c) and (d): HNT/Fe3O4-H sample; (e) and (f): HNT/Fe3O4-SG sample. Magnification: 9 kX (left) and 200 kX (right)

EDS DISTRIBUTION MAPS

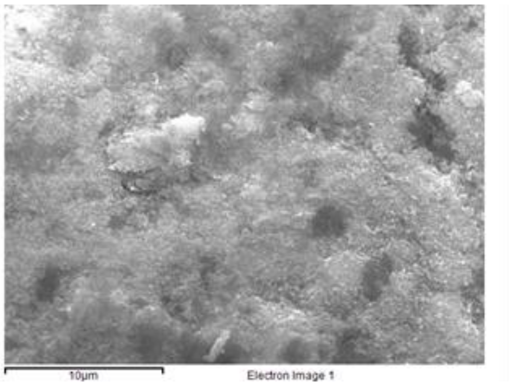

(a)

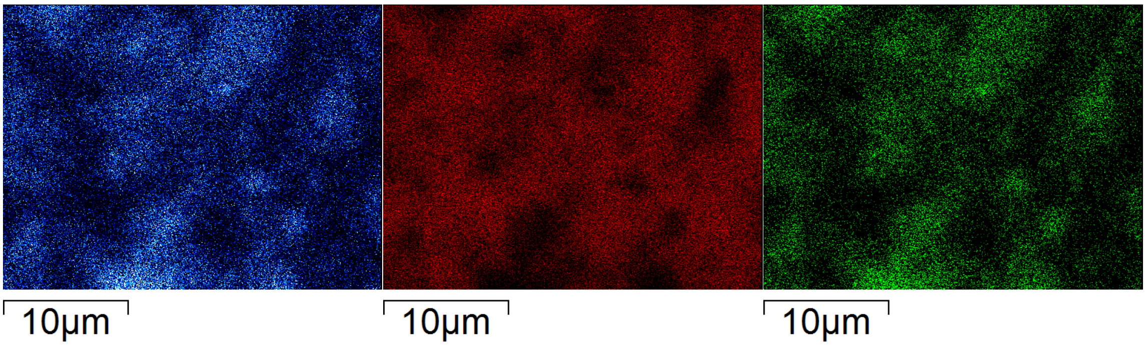

(b) (c) (d)

Figure S3. (a) investigated area and distribution maps of (b) Al, (c) Fe and (d) Si elements of the HNT/Fe3O4-C sample.

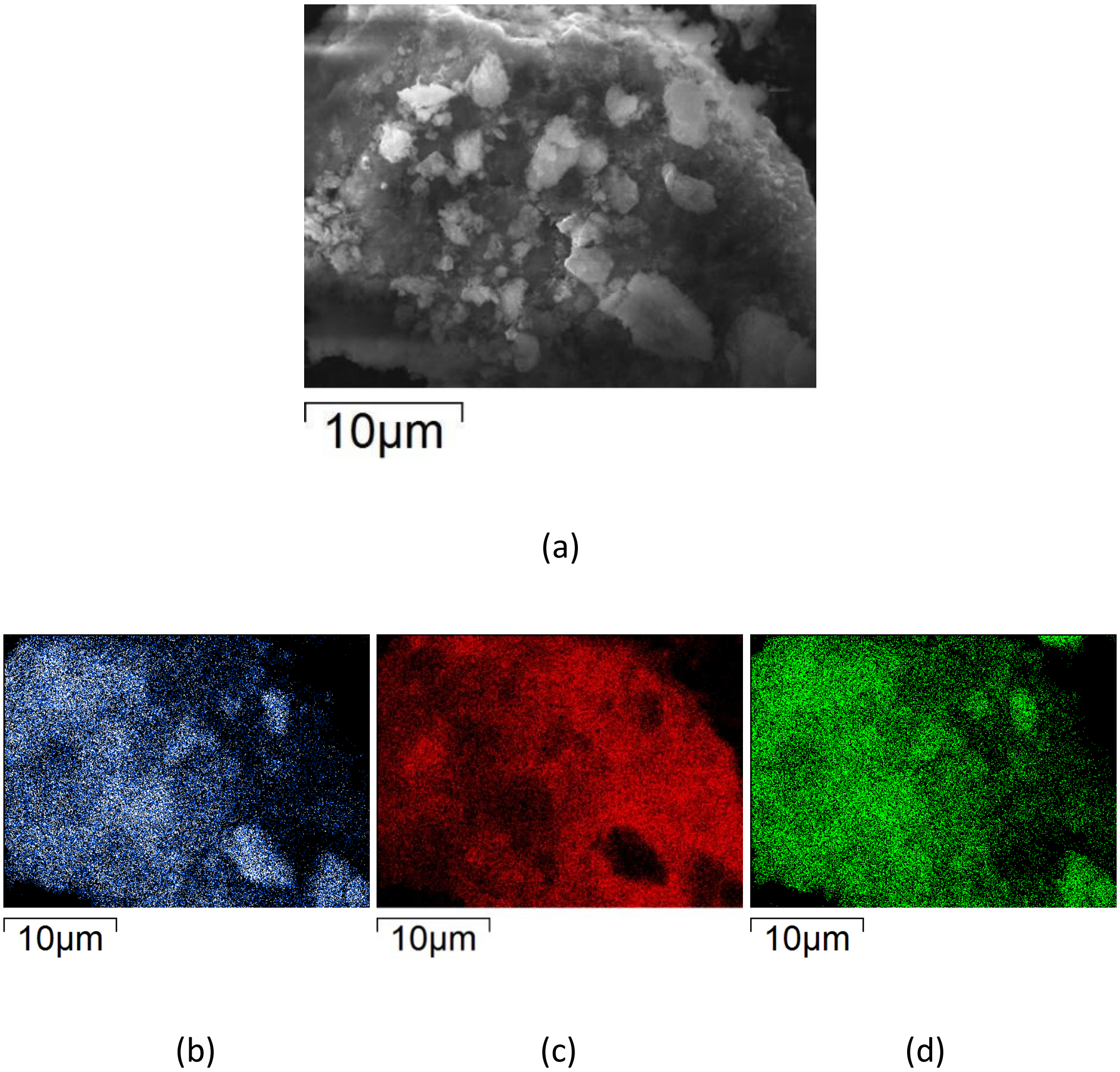

Figure S4. (a) investigated area and distribution maps of (b) Al, (c) Fe and (d) Si elements of the HNT/Fe3O4-H sample.

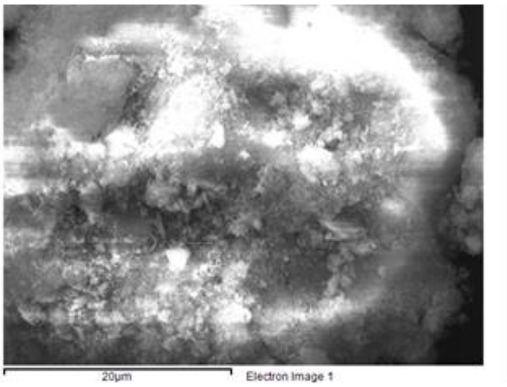

(a)

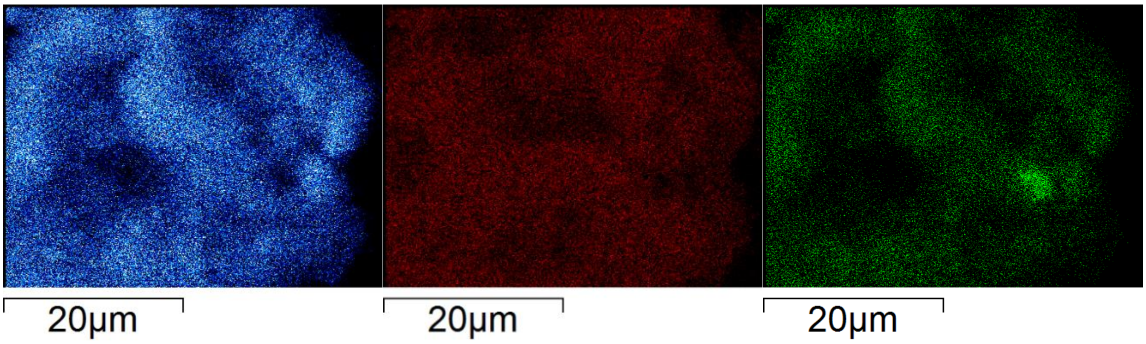

(b) (c) (d)

Figure S5. (a) investigated area and distribution maps of (b) Al, (c) Fe and (d) Si elements of the HNT/Fe3O4-SG sample.

XRPD

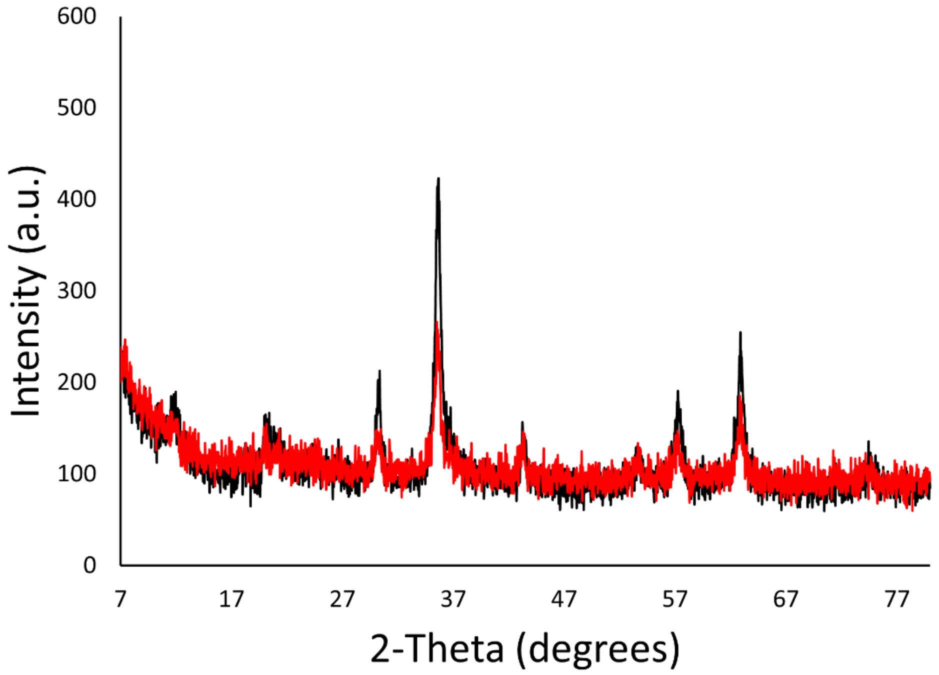

Figure S6. X-ray diffraction pattern of the HNT/Fe3O4-C sample as-prepared (black line) and after three cycles of OFL recover (red line).

The different intensity in the diffraction patterns of the two investigated samples are due to the small amount of recovered sorbent material after usage.

Table S1. Mean particle size and intensity determined by DLS analysis

SAMPLE	mean diameter (nm)	Intensity (%)
HNT	243 ± 25	40.6
	902 ± 98	59.4
Fe4O3-C	164 ± 94	100
HNT/Fe3O4-C	912 ± 80	100
HNT/Fe3O4-SG	905 ± 82	100
HNT/Fe3O4-H	307 ± 36	94.4
	78.8 ± 6.9	5.6

Table S2. Physico-chemical characterization of tap and river water samples, and WWTP effluent

Parameters/Ions		Tap water	River water	WWTP effluent
pH		7.7	7.9	7.3
Conductivity at 20 °C	μS cm^{-1}	278	297	849
Cl^{-}	mg L^{-1}	4.5	3.8	100
NO_3^{-}	mg L^{-1}	0.6	1.5	22.7
SO_4^{2-}	mg L^{-1}	5.0	12.5	49
HCO_3^{-}	mg L^{-1}	195	200	360
Ca^{2+}	mg L^{-1}	38	56	78
Mg^{2+}	mg L^{-1}	12	7.0	17.5
Na^{+}	mg L^{-1}	11	5.0	76.5
